# Reactive Oxygen Species and *H. pylori* Infection: A Comprehensive Review of Their Roles in Gastric Cancer Development

**DOI:** 10.3390/antiox12091712

**Published:** 2023-09-02

**Authors:** Dhiraj Kumar Sah, Archana Arjunan, Bora Lee, Young Do Jung

**Affiliations:** Department of Biochemistry, Chonnam National University Medical School, Seoyang Ro 264, Jeonnam, Hwasun 58128, Republic of Korea; 197784@chonnam.edu (D.K.S.); archanaibms@gmail.com (A.A.)

**Keywords:** antioxidants, gastric cancer, *Helicobacter pylori*, inflammation, oxidative stress

## Abstract

Gastric cancer (GC) is the fifth most common cancer worldwide and makes up a significant component of the global cancer burden. *Helicobacter pylori* (*H. pylori*) is the most influential risk factor for GC, with the International Agency for Research on Cancer classifying it as a Class I carcinogen for GC. *H. pylori* has been shown to persist in stomach acid for decades, causing damage to the stomach’s mucosal lining, altering gastric hormone release patterns, and potentially altering gastric function. Epidemiological studies have shown that eliminating *H. pylori* reduces metachronous cancer. Evidence shows that various molecular alterations are present in gastric cancer and precancerous lesions associated with an *H. pylori* infection. However, although *H. pylori* can cause oxidative stress-induced gastric cancer, with antioxidants potentially being a treatment for GC, the exact mechanism underlying GC etiology is not fully understood. This review provides an overview of recent research exploring the pathophysiology of *H. pylori*-induced oxidative stress that can cause cancer and the antioxidant supplements that can reduce or even eliminate GC occurrence.

## 1. Introduction

Gastric cancer/gastric adenocarcinoma (GC) is a global health threat. It is the third most common cause of cancer-related death and the fifth most common cancer globally, being responsible for approximately 800,000 cancer-related deaths annually [1]. GC is a heterogeneous disease from a morphological and molecular perspective [2] and is also a multifactorial disease that has genetic and environmental etiologies [3]. The American Cancer Society classifies 90–95% of GC cases as adenocarcinomas (arising in gland cells that produce gastric acids and mucus), 4% as lymphomas (arising in the stomach lymph tissues), 1–3% as hereditary diffuse gastric cancer (HDGC), and the remainder as gastrointestinal stromal tumors (in the interstitial cells of Cajal). There are two main types of GC (cardia and non-cardia) depending on their anatomic location. For most, the upper stomach constitutes the cardia subtype, while the mid-distal stomach constitutes the non-cardia subtype. Non-cardia GC is the subtype most commonly caused by chronic Helicobacter pylori (*H. pylori*) infection [4,5]. GC is predominantly caused by a *H. pylori* infection [6]. High GC risk is associated with virulent *H. pylori* strains, smoking, poor diet (e.g., high salt, smoked foods, and low iron), unhealthy lifestyles, and pro-inflammatory host genetic factors [7].

Approximately half of the world’s population is thought to be infected with *H. pylori. H. pylori* infection prevalence and virulence factor genotypes differ extensively depending on geographical regions [8]. *H. Pylori* is a Gram-negative, curved or S-shaped, microaerophilic, and highly motile bacterium because of its unipolar bundle of sheathed flagella [9]. *H. pylori* strains exhibit a distinct population structure congruent with their coevolution with humans, leading to implications about the history of the disease in humans [10]. A contaminated water source can result in infection as it can be cultivated from infected individuals’ vomit, stools, and saliva, and it most commonly spreads through fecal–oral and oral–oral routes [11]. More than 47 species of Helicobacter have been recognized to date and have been classed into two major categories: gastric and non-gastric (enterohepatic) [12,13]. In its ecological niche, *H. pylori* colonizes the deep gastric mucus layer, producing urease, promoting motility and adhesion [14]. The Helicobacter species found in the stomach are *H. pylori*, *H. mustela*, *H. heilmannii*, *H. felis*, *and H. acinonychis*.

It is believed that *H. pylori* is the primary cause of chronic gastritis and can cause other severe gastroduodenal diseases, such as mucosa-associated lymphoid tissue lymphoma (MALT) and gastric and duodenal peptic ulcer disease (PUD), as well as GC [15]. Sustained chronic inflammation impairs and depletes the parietal cells, causing hypochlorhydria and achlorhydria. As acidity decreases, harmful pro-inflammatory gastric microbes colonize in gastric mucosa, thereby secreting genotoxic pro-inflammatory metabolites and carcinogens that directly facilitate stomach cancers [16].

*H. pylori* is closely linked with chronic gastritis and is characterized by generating reactive oxygen species (ROS) and nitric oxide (NO) metabolites and reducing antioxidant mechanisms [17]. A high environmental ROS content favors *H. pylori* growth. Because of its defensive strategies, *H. pylori* can survive, long-term, in the stomach environment despite the inflammatory response of the host. The longer it remains, the more likely *H. pylori*-induced oxidative stress will activate multiple signaling pathways, promoting cancer development. Despite this, exactly how *H. pylori* triggers oxidative stress-mediated gastric carcinogenesis is unclear. In this study, we discuss recent developments in *H. pylori’s* oxidative stress-induced GC and its pathogenic mechanisms as well as the most recent advances in antioxidant treatment strategies for *H. pylori*-induced GC.

## 2. The Role of ROS and Gastric Carcinogenesis

**Figure 1:**
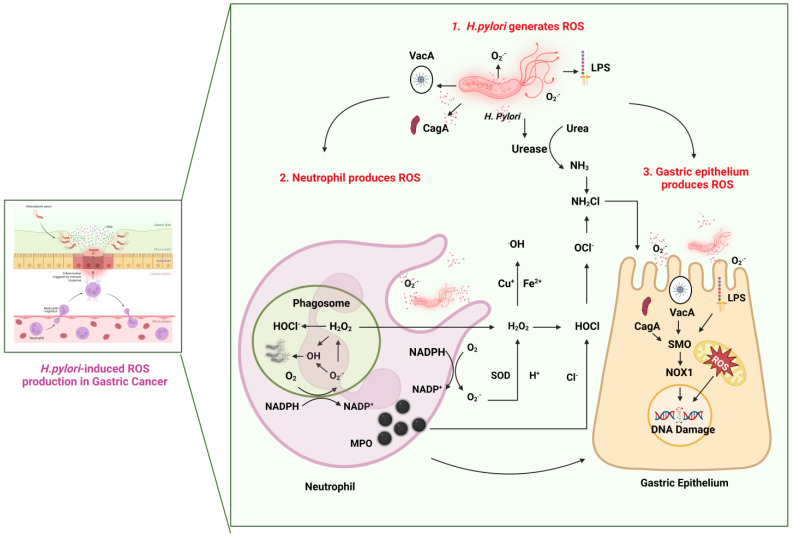
The role of ROS generation in gastric carcinogenesis. The prolonged presence of *H. pylori* in gastric mucosal cells leads to oxidative stress, chronic inflammation, and DNA damage. In addition, *H. pylori* facilitates the production of ROS and RNS using the host inflammation cells in the gastric mucosa. This results in mucosal damage via the activation of neutrophils, which releases the oxidative stressors that facilitate the exposure of gastric epithelium to reactive oxygen species. Even though ROS can be generated by various cells, including macrophages and epithelial cells, neutrophils generate the bulk of ROS. Recent research has indicated that H. pylori-induced ROS production may influence gastric epithelial cell signal transduction, which contributes to GC [23]. (O_2_^•−^: superoxide anion, ^•^OH: hydroxyl radicals, H_2_O_2_: hydrogen peroxide, NH_3_: ammonia, NH_2_Cl: monochloramine, OCl^−^: hypochlorite ion, HOCl: hypochlorous acid, Cl^−^: chloride, H^+^: hydrogen ion, SOD: superoxide dismutase, Cu^+^: cuprous ion, and Fe^2+^: ferrous ion).

Cellular signaling relies on ROS, which are by-products of cellular metabolism. There is a wide variety of molecules with oxidizing properties that can cause oxidative stress [18]. ROS include superoxide anions (O_2_^•−^), hydroxyl radicals (HO•), nitric oxide radicals (•NO), and lipid radicals, where unpaired electrons are present. Other ROS may also have oxidizing properties, but they do not constitute free radicals such as hydrogen peroxide (H_2_O_2_), peroxynitrite (ONOO), and hypochlorous acid (HOCl) [19]. As excess ROS accumulate, they can damage cellular components such as membranes, proteins, and DNA, which can be detrimental to cancerous and noncancerous cells [20]. Various research studies have shown that *H. pylori* infection induces ROS generation and oxidative stress, which can lead to GC [21,22]. In this study, we discuss the pathology of ROS generation in *H. pylori*-mediated gastric carcinogenesis (see Figure 1).

Other studies have shown that *H. pylori* produces a high number of superoxide anions (O_2_^•−^) to inhibit its effect on inflammatory cells. The cytotoxicity of O_2_^•−^ is relatively low; however, hydroxyl radicals (^•^OH) derived from Fenton’s reaction with metals and hydrogen peroxide (H_2_O_2_) are much more toxic, meaning that *H. pylori*-produced O_2_^•−^ may indirectly damage the gastric epithelial cells [24]. During unfavorable conditions, *H. pylori* transforms into a coccid morphology from its normal helical bacillary morphology [25]. In contrast to H. pylori’s helical form, its coccoid form produces more ^•^OH, but how it produces them is unknown [26]. Among the potential sources of ROS and RNS in a stomach infected with H. pylori are neutrophils, gastric mucosal cells, and vascular endothelial cells, and, as mentioned before, the main source of ROS/RNS is believed to be neutrophils [27]. Cell membranes produce ROS by catalyzing nicotinamide adenine dinucleotide phosphate oxidase (NADPH oxidase; NOx).

In gastric cells, neutrophils engulf bacteria and convert them into phagosomes, while NADPH oxidase (NOx) generates ROS to kill bacteria [28]. By donating an electron, cytoplasmic NADPH activates NOx’s catalytic subunit gp91phox. SOD converts O_2_ into H_2_O_2_ by transferring this electron from NOx to molecular oxygen [29]. Toxic and highly reactive ROS are created following H_2_O_2_′s conversion into HOCl by Cl ions in phagocytes. Additionally, H_2_O_2_ combined with the catalysts “Fe^2+^/Cu^+^” yields OH^−^, which is also highly reactive. Neutrophils kill bacteria using ROS, such as HOCl and OH [30,31]. However, despite their ability to eradicate bacteria, ROS cannot completely eliminate them. Consequently, inflammatory mediators are released excessively by the stomach epithelium in an ongoing attempt to kill bacteria [32,33]. Additionally, epithelial cells and neutrophils produce nitric oxide (NO) by expressing inducible nitric oxide synthase (iNOS), while NO promotes peroxynitrite. Thus, NOx and iNOS overexpression results in high oxidative stress caused by ROS/RNS [34]. Continued exposure to high levels of ROS interactions and imbalances of oxidant–antioxidant balances eventually lead to DNA damage in gastric epithelial cells and cell death [18,35].

ROS are produced by gastric epithelial cells in H. pylori-infected stomachs [36]. However, the host combats the H. pylori-induced ROS through two canonical pathways. (1) By reducing molecular oxygen through NADPH, O_2_^•−^ is produced, which is capable of dismutation to produce ROS. Also, (2) spermine is converted back to spermidine by the enzyme spermine oxidase, resulting in the production of H2O2. Despite their potential to adversely affect bacteria’s survival, *H. pylori* has developed numerous ways to combat them. Although *H. pylori* has adapted to its ecological niche, ROS-mediated oxidative DNA damage and mutations may contribute to its survival [37]. The gastric epithelium passively produces ROS as a by-product of mitochondrial respiration when stimulated by bacterial cytotoxic factors of *H. pylori* or cytokines [17]. Recently, phagocytic and non-phagocytic epithelial cells of the alimentary tract expressed NOx [38].

In addition to causing neutrophils to produce O_2_^•−^, *H. pylori* lipopolysaccharides (LPS) stimulate gastric epithelial cells to produce NOx by activating Toll-like receptor 4 (TLR4) on their surface [39,40]. There is also evidence that cytotoxic factors released by H. pylori, such as vacuolating cytotoxins, cytotoxin-associated genes (CagA), and peptidoglycan, promote oxidative stress in gastric epithelial cells [41]. Transfecting gastric epithelial cells with the cagA gene induced significant ROS production in the cells by locating a fraction of the CagA protein to the mitochondria [42]. Additionally, there is evidence that ROS production plays a role in accelerating the cell cycle and subsequent proliferation of cells [43].

Oxidative stress induced by *H. pylori* plays a multifaceted role in the gastric mucosa. *H. pylori* invades human gastric mucosa through neutrophils, which serve as convenient tools for the bacteria to attack the mucosa. Even though its role in gastric epithelial cell signal transduction or carcinogenesis is unclear, H. pylori-induced oxidative stress might also play a significant role in signal transduction.

## 3. The Mechanisms Underlying *H. pylori*-Induced Oxidative Stress

**Figure 2:**
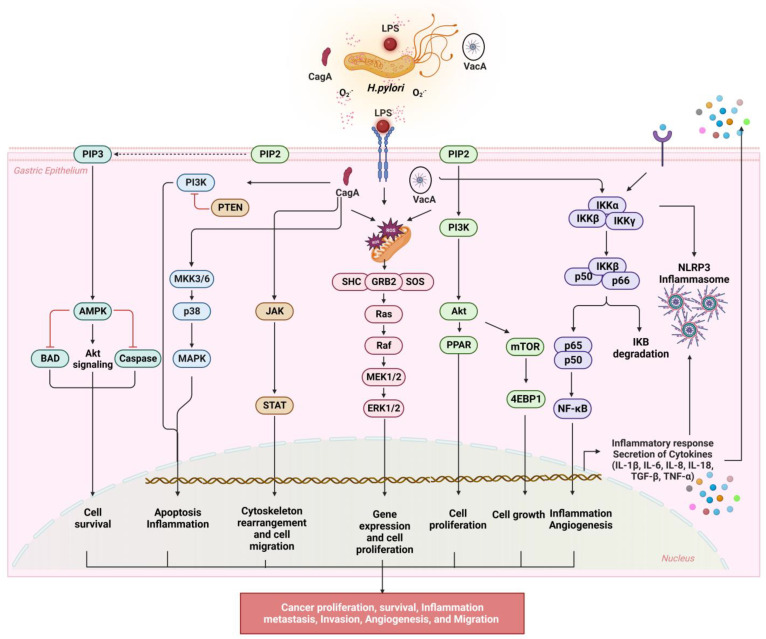
The signaling pathways involved in *H. pylori*-mediated ROS production in gastric cancer. As a result of *H. pylori’s* virulence factors, gastric epithelial cells undergo oxidative stress, which activates the inflammatory signaling pathways NF-κB (involved in inflammation and angiogenesis), PI3K/Akt/mTOR (cell proliferation), AMPK (AMPactivated protein kinase) (cell survival), PTEN/MAPK (apoptosis and inflammation), ERK (gene expression and cell proliferation), JAK/STAT3 (cytoskeleton arrangement and cell migration), and NF-κB-mediated NLRP3 inflammasomes. Consequently, pro-inflammatory cytokines (IL-1β, IL-6, TNF-α, and COX-2) are secreted into the gastric cancer cells, which lead to inflammation, cell cycle arrest, invasion, and migration.

As ROS are generated in cancer cells, we can connect them with signal pathways that stimulate tumor development, as well as distortions in broader signaling networks that lead to cancer progression. Dysregulated signaling interferes with the normal mechanisms of control [44], and this review describes a few signaling pathways implicated in GC caused by oxidative stress mediated by *H. pylori*. In oxidative stress-induced cancers, three pathways are frequently activated: the PI3K/AKT/mTOR pathway, the JAK/STAT3 signal transduction pathway, and the NF-κB/MAPK pathway (see Figure 2).

### 3.1. NF-κB Signaling

The nuclear factor kappa-light-chain-enhancer of activated B cells (NF-κB) plays a prominent role in the expression of inflammation-promoting genes, such as cytokines, chemokines, and adhesion molecules [45]. The transcriptional factor NF-κB orchestrates innate and adaptive immune responses during host responses to microbial infections. NF-κB activity is also associated with the initiation and progression of gastrointestinal cancer as it induces chronic inflammation, cellular transformation, and proliferation [46]. The study confirmed that *H pylori* directly infected transformed gastric epithelial cells to activate NF-κB rapidly (within 30 min), translocate p50/RelA and dimers into the nucleus, and quickly accumulate IL-8 mRNA [47]. The last two decades have seen extensive research conducted on the effects of *H. pylori* carrying a 40 kb gene cluster called the cag pathogenicity island (cagPAI). These cagPAI genes, such as cagE, cagL, and cagI, have been identified as crucial for the response to *H. pylori*-induced NF-κB [48,49,50]. CagPAI is encoded largely via the bacteria’s type 4 secretion system (T4SS), which transports the effector CagA from *H. pylori* into the host cell’s cytoplasm [51]. When the T4SS binds to some plasma membrane receptors of the host cell, an increase in the signaling activity is triggered, which causes the NF-κB to be activated [52]. The T4SS transports peptidoglycans from *H. pylori* into host cells. As a result of the nucleotide-binding oligomerization domain 1 of the peptidoglycan cytoplasmic receptor (NOD1), the serine–threonine kinase RICK and the TNF receptor-associated factor 3 (TRAF3) are activated, which in turn activates IFN regulatory factor 7 (IRF7) in mice [53]. Aside from cagPAI, outer membrane proteins from the Hop- and Hor-gene families, gamma-glutamyl transpeptidase (GGT), and vacuolating cytotoxin A are also found. Hop family members include sialic acid-dependent adhesins (SabA), blood group antigen binding adhesins (BabA), adherence-associated lipoproteins A and B (AlpA and AlpB), HopZ, HopQ, and outer inflammatory proteins (OipA) [54,55]. *H. pylori* adheres to host cells through the binding of HopQ to carcinoembryonic antigen-related cell adhesion molecules (CEACAMs) 1, 3, 5, and 6 [56,57]. T4SS function can be enhanced, and *H. pylori* adherence is significantly supported by these molecules. In gastric epithelial cells, OipA activates NF-B in a cagPAI-independent manner [51,58]. Recent studies have reported that *H. pylori*-induced increases in ROS/NADPH oxidase levels activated the NF-κB pathway in AGS cells [59,60]. In gastric epithelial cells, *H. pylori* activates NF-κB and cytokine expression [61]. Another potential mechanism of *H. pylori* involved in regulation of NF-κB and T4SS–cytokine expression involves the TNFα-inducing protein (Tipα), which interacts with cell surfaces prior to entering gastric cells [62]. *H pylori* induces classical and alternative NF-κB pathways through its effector ADP-l-glycero-β-d-manno-heptose (ADP-heptose) [63]. Ferrand et al. reported that in vitro infection with *H. pylori* induces mesenchymal stem cell migration via an NF-κB-dependent pathway [64]. It has also been proposed that ROS both activate and deactivate the IKK complex, resulting in downstream effects. ROS can activate NF-κB via alternative IκBα phosphorylation, either resulting in or preventing IκBα degradation. Similarly, *H. pylori*-induced NF-κB activation decreased IκBα and induced MMP expression in AGS cells [60]. ROS may also influence the DNA binding properties of NF-κB proteins. A Trx1-dependent process involving Ref-1 is required to reverse the oxidation of p50 on its DNA-binding domain. Conversely, ROS-dependent processes that lead to the phosphorylation of RelA result in greater activation of NF-κB [65].

As mentioned above, *H. pylori*-induced oxidative stress response can trigger NF-κB pathway activation by releasing the virulence factors that activate the upstream kinases (IKK, NIK, and Akt), thereby causing the degradation of IκB or altering the nuclear translocation and transcription factor binding to DNA by modifying the heterodimers of the transcription factor.

### 3.2. NLRP3 Inflammasome Activation

An increasing body of evidence suggests that NF-κB signaling is also involved in the regulation and integration of energy metabolism in addition to triggering inflammation [65]. NF-κB hinders inflammasome activation by eliminating damaged mitochondria [66]. Surprisingly, however, NF-kB appears to be responsible for both priming NLRP-3 inflammasomes and inhibiting excessive inflammation, although the mechanism by which this inhibition occurs is not currently understood [67]. Inflammasomes are multimeric proteins found in the cytosol that assemble in response to perturbations in the cellular environment [68]. Consequently, caspase-1 is activated, which promotes the maturation and release of interleukin (IL)-1β and IL-18, inflammatory cell death, and pyroptosis. These inflammatory cytokines contribute to low-grade systemic inflammation, while aberrant NLRP3 activation can modulate the pathogenesis of inflammation-associated disease in the body [69]. When immune cells, especially macrophages and dendritic cells, are infected with *H. pylori*, the elevated IL-1β production via activation of the NLRP3 inflammasome initiates an inflammatory reaction such as neutrophil infiltration. This decreases gastric acid secretion, helping bacteria colonize and survive in the gastric tissue [70,71]. Li et al. demonstrated that *H. pylori* infection induced the expression of IL-1β/IL-18, which activated the NLRP3 inflammasome and produced ROS in THP-1 cells [72]. As well as activating inflammatory genes, NF-κB appears to play a role in limiting NLRP3 inflammasome activation and IL-1β production [73]. Another study reported that IL-1β overexpression activates myeloid-derived suppressor cells (MDSCs) via the IL-1β/IL-1RI/NF-κB pathway in inflammatory and epithelial cells [74]. In addition, IL-18 overexpression may facilitate the immune escape of GC cells by suppressing CD70 and increasing cancer cells’ metastatic ability by upregulating CD44 and vascular endothelial growth factor (VEGF) [75]. Furthermore, NF-κB inhibits the inflammasome via p62 induction.

When macrophages are activated via different NLRP3 inflammasome activators, NF-κB appears to be able to regulate its inflammation by promoting p62-mediated mitochondrial removal (mitophagy) [76]. Therefore, *H. pylori*-induced oxidative stress activates the NLRP3 inflammasome, increasing IL-1β and IL18 expression and contributing to immune responses, migration, and angiogenesis by activating the VEGF and NF-κB pathways in GC.

### 3.3. PI3K/AKT/mTOR Signaling

Autophagy occurs when enzyme-catalyzed metabolic processes break down proteins and organelles for their macromolecule precursors [77]. In response to changes in ROS levels, the phosphatidylinositol 3-kinase (PI3K) pathway is crucial in regulating autophagy. ROS activate PIK3/AKT/mTOR signaling in cancer. After *H. Pylori* infection, CagA-MET activates PI3K/Akt signaling, which contributes to *H. pylori*-associated chronic gastric proliferation and NF-kB signaling, causing pro-inflammatory responses [78]. It has been shown that the *H pylori* VacA toxin contributes to gastric injury pathogenesis through phosphorylating protein kinase B (Akt) and glycogen synthase kinase-3β (GSK3β) via a PI3K-dependent pathway [79,80]. *H. pylori*-induced PIK3/AKT/mTOR and NF-κB activation decrease IκBα and induce matrix metalloproteinase (MMP) (MMP-7 and -10) expression, invasive phenotypes, and migration in AGS cells [60]. The PI3K pathway is activated by binding RTK/GPCR/GTP-binding proteins to adapter proteins. As a result of this phosphorylation, PIP2 (phosphatidylinositol 3,4-bisphosphate) is converted into PIP3 (phosphatidylinositol 3,4,5-triphosphate). Following its activation, PIP3 activates 3-phosphoinositide-dependent kinase 1 (PDK1) and Akt. Phosphorylated Akt promotes cell survival, proliferation, differentiation, and migration [81]. A change in ROS levels inhibits autophagy, while changing the PI3K catalytic subunit facilitates it. The downstream Akt proteins act against autophagy initiation in response to increases in ROS levels. Also, studies have shown that MMP-7 overexpression correlates significantly with tumor morphology, phenotype, and staging of GI tract tumor progression [82]. A similar diagnostic marker of GC is MMP-10 overexpression [83], which regulates angiogenic and apoptotic pathways to promote tumor progression [84]. Furthermore, by interacting with the mammalian target of rapamycin complex 1 (mTORC1) and arresting autophagic gene expression, Akt inhibits both cell growth and death. AMPK counteracts Akt, while the mTORC1 and mTORC2 proteins inhibit autophagy at moderate levels of ROS [85]. The mTORC1 protein plays a key role in regulating cell survival, growth, proliferation, and metabolism [86]. However, at high levels of ROS, the mTORC2 protein can promote cellular senescence by activating autophagy [87].

In addition to being an inhibitor of the PI3K pathway and possessing pro-autophagic properties, PI3K is negatively regulated by phosphatase and tensin homolog (PTEN) [88]. The PTEN protein dephosphorylates PIP3 and inhibits Akt activation via PIP3 [89]. mTOR is activated because of Akt phosphorylation [90], and mTOR then phosphorylates S6K1 and 4EBP1, which increases ribosome and cell-cycle regulatory protein translation [91]. PI3K/Akt/mTOR plays a vital role in tumor initiation and progression, including proliferative activity and apoptosis. In GC, PI3K signaling has also been linked to metastatic cascades that include proteolytic activity, cytoskeletal remodeling, and chemotherapy resistance. Even though these pathways are currently poorly understood, clinical trials are currently testing the effects of targeting the PI3K/Akt/mTOR pathway.

### 3.4. JAK/STAT Signaling

The cytokine activates cellular signaling pathways including the Janus Kinases (JAK: a family of tyrosine kinases) and the Signal Transducer and Activator of Transcription proteins (STAT) together when it binds to the receptors. This constitutes the JAK-STAT signaling pathway, which is related and evolutionary conserved. Numerous physiological and pathological processes are affected by this pathway, including inflammation, hematopoiesis, and immune responses [92]. *H. pylori*-induced JAK1/STAT3 activation, increased ROS, and NADPH oxidase activation may mediate MCP-1, iNOS, NF-κB, and IL-8 production. [59,61,93]. In contrast, *H. pylori*-induced integrin α5 can mediate cell adhesion and migration by decreasing ROS and suppressing JAK1/STAT3 activation in gastric epithelial cells [94]. Studies have also shown that *H. pylori* activates STAT3 by increasing its Tyr (705) phosphorylation, nuclear localization, binding to DNA, and transcriptional activity. Additionally, ROS produced by *H. pylori*-infected cells increased IL-6 expression and binding to IL-6 receptors [95], while other studies have confirmed that *H. pylori* infection increases IL-6 expression in GC [96,97,98]. Iqra et al. reported that *H. pylori* infection increases the secretion of IL-10, IL-6, and TGF-β, which mediates hyperactivation of JAK/STAT signaling, deactivating the suppressor of the cytokine signaling 1 (SOCS1) gene via hypermethylation of the promoter region in GC [99].

Hongyan et al. found that in GC cells, the STAT3 signaling pathway induces mitogen- and stress-activated protein kinase 1 (MSK1), which activates H3S10 phosphorylation and increases the potential for tumorigenesis [100]. In contrast, the virulence factor CagA induces IL-6 expression by recruiting PKCδ via eEF1A1 in the cytoplasm to increase the phosphorylation of STAT3S727 in the nucleus of AGS cells [101]. Guo and Ding showed that high thioredoxin1 (Trx1) could mediate HP infection pathogenicity via the IL6/STAT3 pathway in GES-1 cells [102]. In these cells with high Trx1 expression, apoptosis was induced, cyclin D1 levels decreased, and p21 levels increased [103]. *H. pylori* caused ROS generation, which increased cytokine expression (IL-6, IL-10, and TGF-β), which reduced the exogenous inhibitors and epigenetic hypermethylation of promoter regions that activated JAK/STAT3 signaling, thus causing inflammation, proliferation, and angiogenesis.

### 3.5. MAPK/ERK/JNK Signaling

Mitogen-activated protein kinase (MAPK) cascades regulate various cellular activities, including inflammation, apoptosis, proliferation, and differentiation. The MAPK family comprises three major subfamilies: extracellular-signal-regulated kinases (ERK), c-jun N-terminal kinases or stress-activated protein kinases (JNK or SAPK), and MAPK [104]. It is known that CagA is dominated by the activation of ERK and JNK subgroups, among the four major branches of ERK, JNK, p38 MAPK, and ERK5—where ERK is responsible for cell differentiation and growth—and Ras/Raf is its upstream signal, while JNK plays an important role in cell apoptosis and inflammation—both of which contribute to the progression of GC as a result of CagA [105]. *H. pylori*-induced increases in ROS and NADPH oxidase activation of MAPK (ERK1/2, JNK1/2, and p38) and MCP-1 in AGS cells [59,106]. We previously mentioned that the adhesion molecules of BabA protein recognize both H-type 1 and Lewis b (Leb) antigens expressed on gastric mucosa leading to the initial step of infection. Subsequently, SabA adhesin mediates *H. pylori* binding to inflamed gastric mucosa by recognizing sialyl-Lewis a (sLea) and sialyl-Lewis x (sLex) antigens to establish persistent colonization [54]. Yang et al. reported that Lewis antigen expression and colonization density are related to MAPK signaling in *H. pylori*-induced gastric inflammation in children and adults [107]. Another study found that phosphorylated CagA triggers ERK/MAPK signaling through interactions with SHP2, C-terminal Src kinase (CSK), and Crk junction protein [108]. An N-SH2 and C-SH2 domain of CagA, as well as two structural domains of SHP2, are required for a complex to be formed between CagA and SHP2. By activating SHP2, Ras/Raf/MEK/ERK signaling pathways are activated, which leads to ERK activation through both RAS-dependent and non-dependent pathways [109]. Activation of MAPKs and AP-1 by *H. pylori* increased gastric epithelial cell invasion and MMP-10 expression by decreasing PPAR-γ-mediated catalase expression and increasing ROS levels [110]. Another study showed that *H. pylori* induced mitochondrial dysfunction and ROS-mediated IL-8 expression by activating PPAR-γ and catalase in gastric epithelial cells [111]. Yakun Bi et al. reported that *H. pylori* infection increased cell viability, CyclinD1 expression, JNK and ERK phosphorylation, and cellular ROS content as well JNK, ERK, and p38MAPK phosphorylation [112]. *H. pylori* is also known to activate ASK1 in gastric epithelial cells as a result of ROS and Cag pathogenicity islands, while ASK1 is also responsible for maintaining sustained JNK activation and apoptosis induced by the pathogen.

In contrast, TAK1 controls *H. pylori*-mediated JNK activation and cytokine production. ROS-mediated apoptosis is regulated via the ROS/ASK1/JNK pathway by inhibiting TAK1 or downstream p38 MAPK; ASK1 suppresses TAK1 and downstream NF-κB [113]. It has also been demonstrated, however, that altered intracellular calcium (Ca^2+^) concentrations are associated with ROS generation through NADPH oxidase activation in macrophages. The calcium signaling pathway in phagocytes plays a crucial role in the activation, migration, and resolution of infection and inflammation [114]. A transient receptor potential melastatin 2 (TRPM2)-deficient macrophage is not able to control intracellular Ca^2+^ levels when stimulated with *H. pylori* and will lead to calcium overload. Furthermore, increased intracellular calcium levels in TRPM2^−/−^ macrophages will exacerbate the activity of MAPK and NADPH oxidase, leading to an increase in gastric inflammation. Similarly, *H. pylori* infection decreased SOD-1 and HO-1 protein levels, SOD activity, and mitochondrial dysfunction by inhibiting the Nrf2 pathway in AGS cells [115].

### 3.6. ROS and Ferroptosis

Recently, research on ferroptosis in cancer has increased markedly, providing a new perspective on cancer therapy. Ferroptosis is iron-dependent cellular death characterized by an accumulation of lipid peroxide within the cell and an imbalance of redox potential [116]. Several small molecules are capable of initiating cell death because of specific molecular events such as apoptosis, ferroptosis, or necrosis [117]. Ferroptosis begins with iron accumulation. High iron levels are linked to lipid peroxidation and abnormal mercaptan iron metabolism, leading to increased ROS production [118]. A transferrin receptor binds to circulating iron as Fe^3+^, allowing it to enter the cell [119]. Through DMT1, iron oxide reductase reduces Fe^3+^ to Fe^2^+, which is then pumped into the iron pool producing ROS. In addition, it contributes to ferroptosis and lipid peroxidation. By contrast, the Xc system simultaneously transports intracellular Glu to the extracellular space and extracellular cystine into the cells for GSH synthesis. Consequently, GPX4 activation causes polyunsaturated fatty acids (PUFAs) to become -OH, which is ultimately a cause of cellular death [116].

SOCS1 is required for p53 activation and ferroptosis in response to aberrant JAK/STAT5 pathway activation [120]. SOCS1 may act as a potential biomarker for uncovering gastric cancer underlying mechanisms. Increasing immunotherapy activity through ferroptosis–immunomodulation may be a viable strategy in GC’s therapy [120]. Furthermore, by promoting Arachidonate 5-Lipoxygenase (ALOX5) expression in pylori-positive GC cells, PHKG2 facilitates ferroptosis induced by (RAS-selective lethal3) RSL3 [121]. It is thought that these findings may contribute to an understanding of the unique pathogenesis of H pylori-induced gastric cancer, as well as provide a means of controlling ferroptosis in diverse situations using genetic, cellular, and immune therapies.

## 4. The Relationship between *H. pylori* Virulence Factors and ROS Production

**Figure 3:**
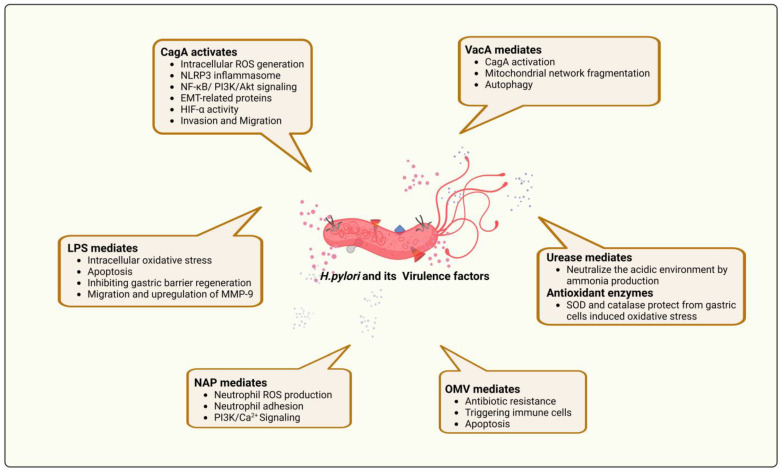
*H. pylori’s* virulence factor in ROS-mediated gastric cancer. The virulence factors of *H. pylori* (CagA, VacA, NAP, OMV, and urease) induce ROS production and inflammatory signaling in host cells and maintain an alkaline environment in which to protect itself against the acidic pH of the host environment. CagA involved in activation of inflammatory pathways, VacA involved in autophagic mechanism, LPS mediates upregulation of cell invasion and migration, NAP involved in neutrophil-mediated inflammation, OMV involved in triggering immune response, and urease/antioxidant enzymes regulating the gastric oxidative stress level.

The virulence of an *H. pylori* infection causes prolonged inflammation in the gastrointestinal mucosa. Multiple virulence factors associated with *H. pylori* strains may contribute to oxidative stress in the host and appear to be a combination of three pathogenic mechanisms: colonization (involving the urease, adhesin, and flagellum chemotactic systems), resistance to immunity (i.e., flagellum, lipopolysaccharides (LPS), VacA, and CagA), and disease induction (DUpA, VacA, and BabA) [122] (see Figure 3). It is also possible to classify these virulence factors as effector proteins. The main effector proteins/toxins released by *H. pylori* include cytotoxin-associated genes (CagA), urease, blood group antigen-binding adhesions (BabA), vacuolating cytotoxins (VacA), outer inflammatory proteins (OIPA), outer membrane proteins, high-temperature requirement A (HtrA), sialic acid-binding adhesins (SabA), outer membrane vesicles (OMV), and neutrophil-activating protein A (NepA) [123].

### 4.1. Cytotoxin-Associated Gene A (CagA)

CagA is one of *H. pylori’s* most studied virulence genes. CagA expression is induced by *H. pylori* adhering to the gastric epithelium [124]. CagA is injected directly into epithelial cells by *H. pylori* through a type IV secretion system [123], whereupon it interacts with host SH2 domains to phosphorylate tyrosine after reaching the host cell. Once this occurs, the cells undergo morphological, apoptotic, proliferation, and motility changes, stimulating the development of GC [125]. Zhan et al. (2022) reported that Hp/CagA^+^ strain infection and pcDNA3.1/CagA vector transfection activated intracellular ROS production and NLRP3 inflammasome and also increased the migration and invasion of GC cells [126]. A study by Jung et al. found that *H. pylori* CagA promoted gastric cell proliferation, ROS production, antiapoptotic activity, cell migration, and invasion by activating the NF-κB and PI3K/Akt signaling pathways and EMT-related proteins [127]. Studies have also shown that in CagA+ *H. Pylori*-infected cells, the CagA protein located in the mitochondria increased HIF-1α activity by causing ROS production via downregulated SIRT3 activity, similar to gastric epithelial cells in hypoxic environments [128].

### 4.2. Vacuolating Cytotoxins (VacA)

Pore-forming toxin vacuolating cytotoxins (VacA) can induce vacuolation in gastric epithelial cells while also inducing apoptosis, inhibiting proliferation, and inhibiting IL-2 release in the gastric epithelium [129]. In gastroduodenal disorders, VacA exerts pleiotropic effects through the activation of specific receptors and can also mediate CagA activation via phosphorylation of Src through receptor-type protein tyrosine phosphatase α (RPTPα) leading to CagA phosphorylation at Tyr972 in AZ-521 cells [130]. In *H. pylori*-infected gastric epithelial cells, Src phosphorylates CagA at its Glu-Pro-Ile-Tyr-Ala (EPIYA-C) motif, which allows CagA to bind to SHP-2 phosphatase [131]. On the other hand, because it is also a critical regulator of mitochondrial fission within cells, VacA induces mitochondrial network fragmentation by recruiting and activating dynamin-related protein 1 (Drp1). As VacA-intoxicated cells undergo mitochondrial fission, Drp1 inhibition suppresses BAX and BCL activation, leading to mitochondrial permeabilization and cell death [132]. Pan Zhu et al. reported that VacA could induce autophagy and increase cell death via numerous dilated numerous endoplasmic reticula (ER), increase the eukaryotic translation initiation factor 2 subunit 1 phosphorylation, and also increase expression of tribbles pseudokinase homolog 3 (TRIB3) upon ER stress in AGS cells [133]. Similarly, increased ROS levels and BECN1, ATG7, and PIK3C3 mRNA expressions were observed following VacA protein treatment in SGC7901 cells, which facilitated a significant decrease in proliferation and an increase in autophagy [134]. Studies have also shown that CagA and VacA can inhibit autophagy signaling upstream along with autophagy-lysosome maturation, inhibiting gastric autophagy in precancerous lesions of GC. Thus, *H. pylori* cannot be effectively eliminated through autophagy due to the persistence of CagA and VacA. Therefore, it will continue to cause inflammation, oxidative stress, apoptosis, glycolysis, and proliferation in precancerous gastric lesions, as well as inflammation, oxidative stress, and apoptosis [135].

### 4.3. Neutrophil-Activating Protein A

Neutrophil-activating protein (HP-NAP) of *H. pylori* is a virulence factor that triggers the neutrophil release of reactive oxygen species during respiratory bursts. HP-NAP stimulates ROS production by neutrophils and increases neutrophil adhesion to endothelial cells [136]. NADPH oxidase generates ROS when HP-NAP is present in neutrophils, and its components are translocated from their cytosol to their plasma membrane. *H. pylori* invades the host gastric epithelium through HP-NAP’s oxidative stress and protects itself from oxidative stress through ROS by forming a biofilm [137,138]. Inducing ROS by HP-NAP occurs via the upregulation of the pertussis toxin (PTX)-sensitive heterotrimeric G protein, PI3K, and SRC family tyrosine kinases, as well as via an increase in calcium concentration in the cell [139].

### 4.4. Other Virulence Factors

Lipopolysaccharides (LPS) inhibit IL-33 expression and activity through oxidative stress, apoptosis, and activation of ERK and sST2, thereby inhibiting IL-33-mediated gastric barrier regeneration [140]. A study by Weronika et al. showed that LPS increase oxidative stress, induce apoptosis, and also induce migration of cells through upregulation of MMP-9 in post inoculation of *H. pylori* in a guinea pig model [141].

The surface of *H. pylori* displays an intact surface membrane with OMV. It is made up of periplasmic proteins, toxins, OMPs, lipids, and extracellular DNA (eDNA) [142]. Stress responses often result in the formation of OMV. Similarly, in a dose-dependent manner, the OMV in *H. pylori* protect against toxic compounds like H_2_O_2_, as well as against an antimicrobial peptide produced by epithelial cells called LL-37 that inhibits bacterial growth. Specifically, OMV enhance bacterial survival, antibiotic resistance, and DNA transfer, triggering immune cells and apoptosis [143].

Numerous stimuli can increase ROS levels in cancer cells. A variety of antioxidant proteins are involved in the process of protecting *H. pylori* against the acidic gastric environment, including peroxiredoxins (Prdx), nitric oxide synthase (NOS), superoxide dismutase (SOD), nuclear factor erythroid 2-related factor 2 (Nrf2), thioredoxin reductase (TrxR), and catalase. Higher levels of antioxidant proteins have also been observed in cancer cells because of increased ROS detoxification. An antioxidant enzyme of 2 Cys, peroxiredoxin 2 (PRDX2), is crucial in scavenging ROS and oxidative stress in cells [144]. Wang et al. found that, in in vivo and in vitro models, *H. pylori* infection activated NF-κB and increased PRDX2 expression. Because of the *H. pylori* infection, PRDX2 knockdown increased ROS production and DNA damage [145]. Thus, by releasing and modulating its virulence factors, *H. pylori* induced oxidative stress and ROS production in gastric cancer, which favored the tumor microenvironment. Moreover, targeting the virulence factor may be an effective therapeutic strategy for treating gastric cancer caused by *H. pylori*.

## 5. The Effectiveness of Antioxidant Supplementation in Preventing Gastric Cancer in *H. pylori*-Infected Individuals

**Figure 4:**
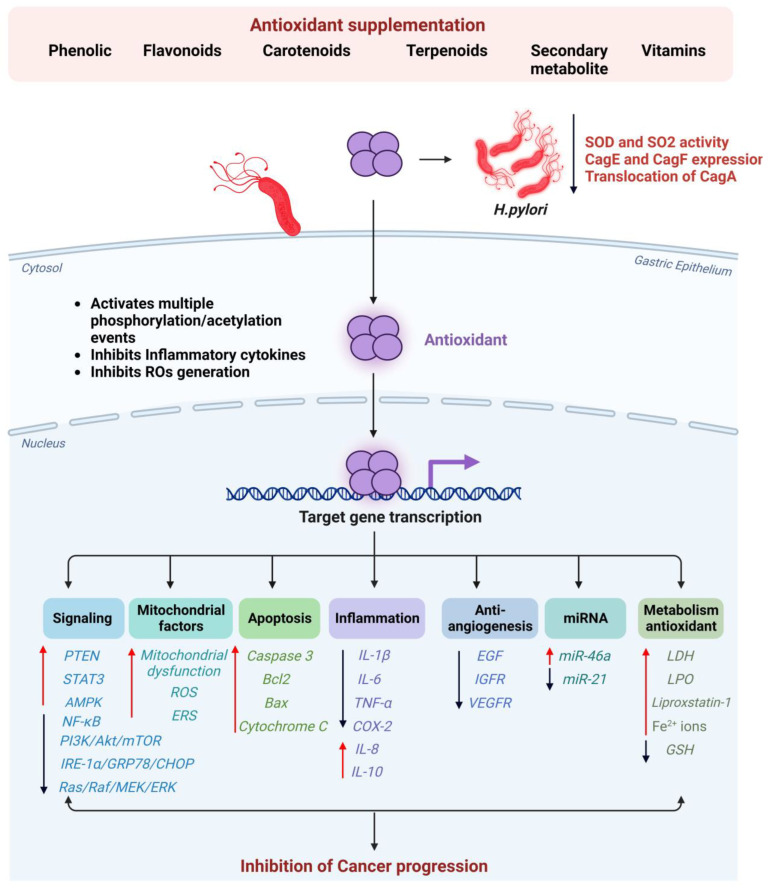
Antioxidant supplementation for *H. pylori*-induced gastric cancer. Various antioxidant-based drugs inhibit signaling pathways that facilitate proliferation, differentiation, metastasis, invasion, and migration. In gastric cancer, these substances reduced oxidative stress, increased antioxidant enzymes, promoted apoptosis, and arrested the cell cycle.

This review summarizes redox homeostasis mechanisms and the relationship between GC and oxidative stress. Additionally, we will discuss recent advances in antioxidant therapy for cancers (see Figure 4). The possibilities and limitations of antioxidant therapeutic strategies in *H. pylori*-mediated GC will also be discussed in the context of several kinds of antioxidant drugs in both in vitro and in vivo studies (see Table 1).

### 5.1. Phenolics

Phytochemicals in plants have been extensively studied by researchers because of their health-promoting potential. Phenolic phytochemicals are a broad category of nutraceuticals found in plants. For example, a phenolic monoterpenoid known as carvacrol is found in the essential oils of plants like oregano (*Origanum vulgare*), wild bergamot (*Citrus aurantium bergamia*), thyme (*Thymus vulgaris*), and pepperwort (*Lepidium flavum*) [176]. The bioactivity of carvacrol includes antioxidant, antimicrobial, and anticancer properties. MNNG (N-methyl-N′-nitro-N-nitrosoguanidine) induces GC in Wistar rats treated with different concentrations of carvacrol, which inhibits oxidative stress, increases apoptotic transcription factors (caspase 3, Bcl2, and BAX), and reduces inflammation [148].

Carvacrol can trigger apoptosis, ROS synthesis, and GSH reduction in AGS cells in a dose-dependent manner [177]. This suggests that carvacrol could be a potential therapeutic treatment for GC and could be a potential candidate for clinical trials in the future. Another important phenolic compound is eugenol (1-allyl-4-hydroxy-3-methoxybenzene), which is mainly derived from *Syzygium aromaticum*. The growing body of literature indicates that eugenol is an antioxidant, anti-inflammatory, antimutagenic, antigenotoxic, and anticancer agent. Efficacious against resistant *H. pylori* strains, eugenol essential oil (EEO) exhibits antibiofilm and anti-inflammatory properties, suggesting it may be a natural alternative to antibiotic therapy [178]. (-)-Epigallocatechin-3-O-gallate (EGCG), a primary green tea polyphenol, is an iron scavenger and an antioxidant, and it also has anticarcinogenic properties [45]. Jing et al. reported that treatment with EGCG inhibited IL-1β, TNF-α, COX-2, and iNOS expression in the gerbil model of *H. pylori*-induced inflammation [165]. Similarly, Jeong et al. (2016) mentioned that the combination of green tea and *Artemisia* extract significantly reduced COX-2, TNF-α, IL-6, and lipid peroxide expression and activated STAT3 relevant to *H. pylori* infection [166].

### 5.2. Flavonoids

The flavonoid phytochemical luteolin induces apoptosis, initiates cell cycle arrest, and inhibits angiogenesis, metastasis, and cell proliferation during carcinogenesis [179]. Stutellarin (Scu) is a flavonoid obtained from *Erigeron breviscapus* (Vant.). Scutellarin (20 and 80 μmol/L) suppressed proliferation and promoted LDH release and apoptosis by raising BAX and Cytochrome C levels and reducing Bcl-2, Wnt1, cytoplasmic β-catenin, and basal β-catenin [146]. The polymethoxylated flavonoid nobiletin, which is present in citrus fruits, possesses a variety of pharmaceutical properties, including anticarcinogenic, anti-inflammatory, antioxidative, and antimetabolic properties [180]. Nobiletin triggers ER stress by activating the IRE-1α/GRP78/CHOP axis, which reduces neutral lipid accumulation, inducing apoptosis and inhibiting GC cell progression [181]. Luteolin is a flavone naturally found in its glycosylated structure in many edible fruits, vegetables, and herbs (e.g., carrots, peppers, peppermint, and oregano) [182]. Iwoana et al. demonstrated that 30 µM of luteolin significantly induced IL-8, IL-10, and NF-κB expression and reduced ADAM-17, MUC1, GalNAcα-R (Tn antigen), and NeuAcα2-3Galβ1-3GalNAc-R (sT antigen) expression in *H. pylori*-infected CRL-1739 cells [154].

### 5.3. Terpenoids and Carotenoids

The Chinese herbal medicine *Danshen* (*Salvia miltiorrhiza Bunge*) contains a pharmacologically active component, Tanshinone IIA (Tan IIA), which has been proven to prevent and treat cancers of the respiratory, circulatory, and digestive systems [183]. In gastric AGS cell lines, Tan IIA was shown to suppress the expression of epidermal growth factor receptor (EGF), insulin-like growth factor receptor (IGFR) by inhibiting PI3K/Akt/mTOR signaling [184], and vascular endothelial growth factor receptor (VEGFR) and to inhibit the Ras/Raf/MEK/ERK pathways [185]. The oxycarotenoid orange-red pigment astaxanthin (ASX) is naturally found in seafood [186] and has been shown to suppress *H. pylori*-induced SOD2 levels and SOD activity and also inhibits mitochondrial ROS [163] by activating PPAR-γ and catalase expression [111].

### 5.4. Other Phytochemical Compounds

A plant triterpene known as Celastrol inhibits the proliferation of GC cells and induces apoptosis. Celastrol directly binds to PRDX2, which leads to increased ROS production, mitochondrial dysfunction, and apoptosis through ROS-dependent stress in the ER [157]. In xenografted GC mice, Celastrol significantly elevated AMPK phosphorylation and activated apoptosis, autophagy, and mTOR following a reduction in Akt, mTOR, and S6K phosphorylation [171]. Additionally, Celastrol was shown to inhibit GC viability by reducing IKB phosphorylation, as well as nuclear P65 protein level, by upregulating miR-146a expression [173]. Furthermore, Celastrol increased PTEN phosphorylation and decreased PI3K, AKT, p65, and IκBα phosphorylation by downregulating miR-21, which in turn, inhibited cell proliferation, migration, and invasion and induced apoptosis and G2/M cell cycle arrest in MKN45 cells [161]. Polyphyllin I (PPI) treatment inhibited GC growth by increasing intracellular ROS/LPO, liproxstain-1, and Fe^2+^ ions and decreasing NRF2 and ferritin heavy chain 1 (FTH1) through NRF2/FTH1 pathway regulation [187]. Geraniol prevents apoptosis, ROS, and cytotoxicity by depleting malondialdehyde levels and reducing reactive DNA damage and nuclear fragmentation. Geraniol significantly reduced the expression of phosphorylated p38 MAPK, ERK1, c-JNK, TNF-α, IL-6, and COX-2 and increased the expression of the antioxidant protein Prdx-1 in *H. pylori*-infected cells [147]. There is a growing body of evidence that *Ganoderma lucidum* can serve as a chemopreventive agent as well as a functional food. The mushroom is known for its medicinal properties, and some extracts of *Glucidum* have shown promising antitumor properties. Inhibition of cell growth and cell cycle via methanolic extracts of the *Ganoderma lucidum* fruiting body were observed in a gastric cancer cell line [175]. In SGC-7901 human gastric cancer cells, recombinant Lz-8 induces autophagy via endoplasmic reticulum stress [188].

Iwona et al. demonstrated that when AGS cells were treated with phenolics (phenolic acid or p-coumaric acid) and flavonoids (kaempferol, astragalin, or tiliroside) at 80 and 160 µM, the mRNA expression of MUC1, ppGalNAcT2, and C1GalT1 was inhibited, and the protein expression of ST6GalNAcT2 and FUT4, C1GalT1, St3Gal-IV, Tn and sialyl T antigen, and MUC1 domain in *H. pylori*-infected AGS cells was also reduced [189]. In traditional Indian medicine, curcumin, which is a secondary turmeric metabolite, has been used to treat gastrointestinal ailments including gastric dyspepsia. As well as modulating apoptosis and cell proliferation, curcumin affects the immune system [190] by attenuating the mRNA expression of the *H. pylori* virulence genes CagE and CagF. It also inhibits the translocation and phosphorylation of CagA in gastric epithelial cells. *H. pylori* strains isolated from mice treated with dietary curcumin could not effectively induce cSrc phosphorylation and IL-8 gene expression [191]. Biocompatible co-polymer PLGA nanoparticles encapsulated with curcumin have also been shown to enhance anti-gastric cancer and anti-*H. pylori* activity [192]. On the other hand, as an antioxidant, Piperine, a nitrogenous substance abundant in black pepper, plays a variety of roles in the metabolism of lipids and drugs, the bioavailability of drugs, antimutagenic effects, and tumor inhibition in the gastrointestinal system [193,194]. In GC cells, Piperine treatment inhibits the adhesion of *H. pylori* by suppressing the expression of the flagellar hook gene flgE and the integral membrane component of the export apparatus gene flhA [195]. It was found that the antioxidant-rich phytochemicals mentioned above and their compounds reduce the effects of oxidative stress induced by *H. pylori* on gastric cancer cell proliferation, angiogenesis, metastasis, and invasion.

### 5.5. Nanosystems in H. pylori-Induced Gastric Cancer

Currently, biomaterials are mainly used as delivery systems for drugs to eradicate *H. pylori*, thus increasing the efficiency of drug delivery. In addition to encapsulating antibacterial agents, biomaterials (including lipid nanoparticles, chitosan nanoparticles, and inorganic nanoparticles) have been used directly in *H. pylori* treatment due to their inherent antibacterial properties. Biomaterials have been demonstrated to be effective in treating *H. pylori* infections in these recent studies.

As a result of their remarkable therapeutic potential, selenium nanoparticles (SeNPs) have earned a high reputation. Biogenic selenium nanoparticles (PG-SeNPs) produced using pomegranate peels (PP) aqueous extract significantly reduced gastric cancer cell viability in a dose-dependent manner [196]. An inorganic nanoparticle is a small particle with a high surface area and many surface-active centers. This characteristic gives them a strong catalytic ability and means that they can be employed in a variety of applications. They are found in inorganic salts (such as silver, gold, and zinc) or oxides with nanoscale sizes (1–100 nm). Metal ion release and ROS production are the main mechanisms by which inorganic nanoparticles eliminate *H. pylori* as an effective remedy [197,198,199,200].

## 6. Future Directions for ROS and *H. pylori*-Related Gastric Cancer Research

The low malignancy stage of GC is relatively asymptomatic, which means that many cases are only diagnosed at an advanced stage. There are still too many unsatisfactory outcomes, and recurrence rates are still high, despite substantial advances in diagnosis and therapeutic strategies and significant improvements in patient survival. Because of its complex pathological microenvironment in vivo, clinical therapies to eradicate *H. pylori* have been difficult to develop. Also, because of its virulence factors, *H. pylori* infection causes prolonged inflammation in the gastrointestinal mucosa. This response triggers chronic oxidative stress, which is responsible for destroying the bacteria through the immune system.

This review has outlined the molecular mechanisms of oxidative stress induced by *H. pylori*-induced GC and how ROS are regulated. In addition, it has identified critical factors that impact signaling pathways. The review has also suggested that targeted therapy against oxidative stress combined with antioxidant supplementation might be a promising way of delaying or even preventing future gastric mucosal diseases resulting from *H. pylori*-induced GC. Further research is necessary to understand better and characterize ROS-mediated *H. pylori* infection and the mechanisms by which they are linked to GC.

## Figures and Tables

**Table 1:** Therapeutic applications of antioxidant supplementation for *H. pylori*-induced gastric cancer.

No.	Antioxidant Supplementation	Model	Inference	References
1	Scutellarin (20 and 80 μmol/L)	AGS, HGC-27, and GES-1 cell lines	SCU suppressed gastric cancer cell proliferation and increased apoptosis by inhibiting the Wnt/β-catenin pathway.	[146]
2	ASX (1 or 5 µM) for 3 h	*H. pylori*-infected AGS cells	ASX inhibited *H. pylori*-induced integrin α5-mediated cell adhesion and migration by decreasing ROS levels and suppressing JAK1/STAT3 activation.	[94]
3	Geraniol (30–100 μM)	*H. pylori*-infected GES-1 cells	Increased the expression of peroxiredoxin-1 (Prdx-1) in GES-1 cells.	[147]
4	Carvacrol (10, 25, 50, and 100 mg/kg BW)	MNNG-induced GC (200 mg/kg BW)/60 days/Wistar albino rats	High doses of carvacrol (50 and 100 mg/kg BW) increased oxidative stress, inflammation, and apoptosis.	[148]
5	SCU (10, 20, and 30 mg/kg)	MNNG-induced gastric carcinogenesis/AGS cell line and Wistar albino rats	A reduction in LDH activity, ulcer index, pH, mucus weight, and percentage inhibition of ulcers was observed after SC treatment.	[149]
6	Korean red ginseng extract (RGE) (0.01–1μg/mL) for 1 h	*H. pylori*-infected AGS cells	RGE treatment decreased IL-8 production, mitochondrial dysfunction, and ROS production by activating Nrf2, inducing SOD-1 and HO-1, and decreasing ROS levels.	[115]
7	ASX (1 or 10 µM) for 3 h	*H. pylori*-infected AGS cells	ASX suppressed MMP expression, cell invasion, and migration via inhibition of PI3K/AKT/mTOR/NF-κB signaling.	[60]
8	Curcumin (5 mM and 20 mM)	*H. pylori*-infected AGS cells	Curcumin treatment inhibited the vacuolation activity of *H. pylori.*	[150]
9	Phycocyanin (150 µM)	*H. pylori-infected* AGS cells	Phycocyanin inhibited AGS cell hyperproliferation by regulating ROS/MAPK signaling pathways and reducing c-myc and CyclinD1 expression.	[112]
10	SCU (0–80 µM)	MGC-803 and AGS	SCU inhibited GC growth and EMT by regulating the PTEN/PI3K pathway.	[151]
11	Silibinin (20 mg/kg or 200 mg/kg) for 4 or 8 weeks	*H. pylori*-infected C57BL/6 mice/ MKN-1 cell line	Silibinin suppressed *H. pylori* infection by inhibiting COX2 and inducing iNOS expression by suppressing NF-κB and STAT pathways.	[152]
12	β-carotene (0.1 or 0.2 µM) for 2 h	*H. pylori*-infected AGS cells	β-carotene suppressed MAPK-driven MMP-10 expression and cell invasion by promoting PPAR-γ-mediated catalase expression and inhibiting ROS levels.	[110]
13	Vicenin-2 (40 µM)	*H. pylori*-infected GES cells	Vicenin-2 enhanced Nrf2 and PTEN in GES cells.	[153]
14	Luteolin (30 µM)	*H. pylori*-infected CRL-1739 cells	Luteolin significantly induced IL-8, IL-10, and NF-κB expression and reduced ADAM-17 expression.	[154]
15	Astragalin (0–40 µM)—6 h	HGC-27, MGC-803, and MKN-28 cell lines	Astragalin-induced apoptosis inhibited the migration and invasion via inhibition of the PI3K/AKT signaling pathway.	[155]
16	Eugenol (0–240 ug/mL)	AGS cell lines	Eugenol inhibited the TGF-β/SMAD4 signaling pathway in GC.	[156]
17	Celastrol (5 mM for 1 h)	SGC-7901 and BGC-823 cell lines	Increased cellular ROS levels led to ROS-dependent endoplasmic reticulum stress, mitochondrial dysfunction, and apoptosis.	[157]
18	Tanshinone IIA (2 and 4 4 μM)	BGC-823 and NCI-H87	Tan IIA upregulated p53 expression and lipid peroxidation (LPO); ferroptosis downregulated xCT expression, intracellular glutathione (GSH), and cysteine levels..	[158]
19	Nobiletin	*H. pylori*-infected GES-1 cells.	Nobiletin significantly decreased the expression of TNF-α, IL-6, COX-2, PI3K, AKT, and MAPK molecules, including p38 and c-Jun amino-terminal expression in *H. pylori*-infected GES-1 cells.	[159]
20	Zeaxanthin (100 μM for 24 h)	AGS, KATO-3, MKN-28, MKN-45, NCI-N87, YCC-1, YCC-6, YCC-16, SUN-5, SUN-216, SUN-484, and SUN-668 cell lines	Zeaxanthin against GC by inhibiting the ROS-mediated MAPK, AKT, NF-κB, and STAT3 signaling pathways.	[160]
21	Celastrol	MKN45 cells	Celastrol inhibited proliferation, migration, and invasion and inactivated PTEN/PI3K/AKT and NF- κB signaling pathways in MKN45 cells by downregulating miR-21.	[161]
22	Celastrol (0 or 2 μM)	HGC27 and AGS cells	Celastrol activated RIP1/RIP3/MLKL pathways and suppressed the level of pro-inflammatory cytokines by downregulating biglycan (BGN) in HGC-27 and AGS cells.	[162]
23	ASX (1 or 5 μM) for 3 h	*H. pylori*-infected AGS cells	ASX inhibited the reduction in mitochondrial ROS caused by *H. pylori* and decreased SOD2 and SOD activity.	[163]
24	ASX (1 or 5 μM) for 3 h	*H. pylori*-infected AGS cells	Astaxanthin inhibited *H. pylori*-induced mitochondrial dysfunction and ROS-mediated IL-8 expression by activating PPAR-γ and catalase.	[111]
25	Ebselen (0 or 100 μM)	AGS and MGC-803 cells	Ebselen may inhibit ROS production triggered by *H. pylori* LPS treatment via GPX2/4 instead of TLR4 signaling and reduce phosphorylation of p38 MAPK, resulting in altered production of IL-8.	[164]
26	α-lipoic acid (10 and 20 µM for 2 h)	*H. pylori*-treated AGS cells	α-lipoic acid inhibited ROS levels, IL-8 expression, activation of MAPK, ERK1/2, JNK1/2, p38, JAK1/2, STAT3, and NF-κB signaling pathways.	[59]
27	Epigallocatechin Gallate (EGCG) (0.05% EGCG in drinking water)	*H. pylori*-infected Mongolian gerbils	EGCG inhibited the IL-1β, TNF-α, COX-2, and iNOS in the gerbil model of *H. pylori*-induced inflammation.	[165]
28	Artemisia and/or green tea extracts	*H. pylori*-infected and high-salt-diet-administered C57BL/6 mice	Artemisia and/or green tea extract treatment significantly decreased the expressions of COX-2, TNF-α, IL-6, lipid peroxide, and activated STAT3 relevant to *H. pylori* infection.	[166]
29	Curcumin	*H. pylori*-infected 8-week-old BALB/c mice	Curcumin reduced the LPO, MPO level, urease activity, the number of colonized bacteria, levels of anti-*H. pylori* antibodies, biofilm formation, IFN-γ, IL-4, gastrin and somatostatin levels in serum, and minimum inhibitory concentration.	[167]
30	Nobiletin (0–50 µM)	SNU-16 cells	Nobiletin-induced apoptosis in SNU-16 cells was mediated via intracellular ER stress-mediated protective autophagy.	[168]
31	Eugenol (0.1–1.7 mM)	AGS cells	Eugenol induced apoptosis (caspase 3 and caspase 8) in the presence of as well as in the absence of functional p53.	[169]
32	α-LA (10 μM and 20 μM) 2 h	*H. pylori*-infected AGS cells	α-LA inhibited NADPH oxidase and ROS production, inhibition of NF-κB and AP-1 activation, induction of oncogenes, β-catenin nuclear translocation, and hyperproliferation in AGS cells.	[170]
33	Celastrol	AGS and YCC-2 cells GC xenografted mice	Celastrol induced apoptosis and autophagy in gastric cancer cells.	[171]
34	RGE (at various concentrations)	*H. pylori*-infected AGS cells	RGE inhibited the expression of MCP-1 and iNOS by suppressing the activation of NADPH oxidase and Jak2/Stat3 signaling.	[93]
35	Catechins (CAs), sialic acid (SA) combination of CA and SA (CASA)	*H. pylori*-infected AGS cells and BALB/c mice	CASA attenuated the caspase-1-mediated epithelial damage.	[172]
36	Celastrol (0–5 µM)	BGC-823, MGC-803, and SGC-7901 cells	Celastrol induced apoptosis by inhibiting NF-κB activity by upregulating miR-146a expression.	[173]
37	Diphenyleneiodonium (DPI) (2.5 or 5.0 μM) 2 h	*H. pylori*-infected AGS cells	DPI inhibited *H. pylori*-induced activation of MAPKs and MCP-1 expression in AGS cells.	[106]
38	Sporoderm-removed spores of *G. lucidum* (RSGLP)	*H. pylori*-infected AGS cells	RSGLP is more effective at inhibiting gastric cancer cell viability and may serve as a promising autophagy inhibitor for gastric cancer.	[174]
39	Resveratrol (100 mg/kg body weight/day) orally for six weeks	Male Kunming mice	Resveratrol inhibited oxidative stress and inflammation in H. pylori-infected mucosa via the suppression of IL-8, iNOS, and NF-κB and the activation of the Nrf2/HO-1 pathway.	[175]

## Data Availability

Not applicable.

## Abbreviations

AP-1: Activator Protein-1; α-LA: α-lipoic acid; ASX: Astaxanthin; BW: Body weight; CagA: Cytotoxin-associated gene A; Drp1: Dynamin-related protein 1; ERK: Extracellular signal-regulated kinase; FTH1: Ferritin Heavy Chain 1; GC: Gastric adenocarcinoma/Gastric cancer; GSH; Glutathione; H. pylori: Helicobacter pylori; HDGC: Hereditary diffuse gastric cancer; HO-1: heme oxygenase-1; •OH: Hydroxyl radical; H2O2: Hydrogen Peroxide; JNK: c-Jun N-terminal kinase; LPO: Lipid peroxidation; LPS: Lipopolysaccharide; MALT: Mucosa-associated lymphoid tissue lymphoma; MAPK: A mitogen-activated protein kinase; MNNG: N-methyl-N’-nitro-N-nitrosoguanidine; NADPH: Nicotinamide adenine dinucleotide phosphate; NF-κB: nuclear factor kappa-light-chain-enhancer of activated B cells; NLRP3: Nucleotide-binding oligomerization domain-like receptor (NLR) family, pyrin domain-containing 3; NO: Nitric oxide; iNOS: Nitric oxide synthase; Nrf2: Nuclear factor erythroid-2-related factor 2; PPI: Polyphyllin I; PTEN: Phosphatase and tensin protein homolog; PI3K: Phosphoinositol 3-kinase; PUD: Peptic ulcer disease; PRDX2: Peroxiredoxin 2; RGE: Korean red ginseng extract; ROS: Reactive oxygen species; RNS: Reactive nitrogen species; Scu: Scutellarin; STAT3: SOD; O2•^−^: Superoxide anion; TLR4: Toll-like receptor 4; VacA: Vacuolating cytotoxins; and VEGF: Vascular endothelial growth factor.
